# Impact of Hyperhomocysteinemia on Valve Calcification and Prognosis in Rheumatic Mitral Valve Surgery

**DOI:** 10.1155/cdr/5833541

**Published:** 2025-12-12

**Authors:** Songhao Jia, Peiyi Liu, Maozhou Wang, Hongkai Zhang, Tingting Liu, Xian Yang, Ruihan Jia, Xiaoyan Hao, Xiaohan Zhong, Meili Wang, Wei Luo, Yihua He, Lei Xu, Xu Meng, Hongjia Zhang, Wenjian Jiang

**Affiliations:** ^1^ Department of Cardiac Surgery, Beijing Anzhen Hospital, Capital Medical University, Beijing, China, ccmu.edu.cn; ^2^ Beijing Laboratory of Cardiovascular Precision Medicine, Beijing Municipal Education Commission, Beijing, China; ^3^ Key Lab of Medical Engineering for Cardiovascular Disease, Ministry of Education, Beijing, China, moe.edu.cn; ^4^ Laboratory for Clinical Medicine, Capital Medical University, Beijing, China, ccmu.edu.cn; ^5^ Department of Radiology, Beijing Anzhen Hospital, Capital Medical University, Beijing, China, ccmu.edu.cn; ^6^ Echocardiography Medical Center, Beijing Anzhen Hospital, Capital Medical University, Beijing, China, ccmu.edu.cn; ^7^ Department of Physiology & Pathophysiology, School of Basic Medical Sciences, Capital Medical University, Beijing, China, ccmu.edu.cn; ^8^ Center for Coronary Artery Disease, Department of Cardiology, Beijing Anzhen Hospital, Capital Medical University, Beijing, China, ccmu.edu.cn

**Keywords:** hyperhomocysteinemia, mitral valve calcification, prognosis, rheumatic heart disease

## Abstract

**Objective:**

Hyperhomocysteinemia is a risk factor for cardiovascular disease, but its impact on valve disease is lacking in research. This study was aimed at investigating the impact of hyperhomocysteinemia on rheumatic mitral valve calcification and prognosis in patients undergoing surgery.

**Methods:**

This study included 672 patients with severe rheumatic mitral valve stenosis who underwent surgery between January 2016 and December 2022. Patients were stratified by preoperative homocysteine levels. Mitral valve pathology was assessed by echocardiography and coronary CTA, with all‐cause mortality as the primary mid‐term endpoint.

**Results:**

Among this surgical cohort of 672 patients with severe rheumatic mitral stenosis, 208 (31.0%) patients were identified with hyperhomocysteinemia. Imaging assessment revealed that these patients had a higher Agatston score for mitral valve calcification (37.47 vs. 17.19, *p* = 0.038) after adjusting baseline data. Restricted cubic splines revealed a significant dose–response relationship, with mitral valve calcification increasing progressively with higher homocysteine levels (*p* < 0.001). The Kaplan–Meier survival analysis showed that patients with hyperhomocysteinemia had significantly lower mid‐term survival rates (log‐rank *p* = 0.004). Through univariate and multivariate COX regression analyses, it was found that hyperhomocysteinemia was an independent risk factor affecting mid‐term postoperative survival (HR, 2.257; *p* = 0.048).

**Conclusions:**

In patients undergoing surgery for rheumatic heart disease, hyperhomocysteinemia was associated with the formation of rheumatic mitral valve calcification and increased mid‐term postoperative mortality.

**Trial Registration:** Chinese Clinical Trial Registry identifier ChiCTR2200067151

## 1. Introduction

Rheumatic heart disease (RHD) is one of the most common cardiovascular diseases, with approximately 41 million patients worldwide [1, 2]. RHD often affects the mitral valve, causing mitral stenosis [3]. In the study of rheumatic mitral valve disease, it was found that 59% of patients would experience mitral valve calcification, which means that the valve disease was more severe and would significantly increase the difficulty of surgery, affecting the prognosis of patients [4–6]. Therefore, due to the high incidence rate of rheumatic mitral valve disease and the harm of mitral valve calcification, it is significant to explore the risk factors that can be intervened.

Hyperhomocysteinemia (HHcy) was first found to be associated with abnormalities of the nervous system and cardiovascular systems in the 1960s. It has been confirmed to be associated with inflammation and atherosclerosis and is an independent risk factor for stroke, myocardial infarction, and heart failure [7, 8]. Recently, the MESA cohort study further demonstrated a significant association between HHcy and the progression of vascular calcification [9], providing an important clue for exploring its role in valvular calcification. However, the relationship between HHcy and rheumatic mitral valve calcification remains largely unexplored. On the other hand, basic research has shown that folic acid supplementation—an intervention commonly used to lower homocysteine levels—can effectively inhibit the development of aortic valve calcification [10]. This mechanistic link led us to hypothesize that HHcy may be associated with the formation of rheumatic mitral valve calcification and adverse patient prognosis.

This study quantitatively assessed rheumatic mitral valve calcification using a multimodality imaging evaluation method, in order to explore the correlation between HHcy and the formation of rheumatic mitral valve calcification in the surgical cohort, and further investigate its relationship with surgical prognosis in patients with rheumatic mitral valve disease.

## 2. Methods

### 2.1. Patients and Study Design

Patients with severe rheumatic mitral valve stenosis (mitral valve orifice area ≤ 1.5 cm^2^ on preoperative echocardiographic short‐axis planimetry) who underwent surgical procedures at the Valve Surgery Center of Beijing Anzhen Hospital, Capital Medical University, from January 2016 to December 2022 were included. Patients with missing imaging data and laboratory test results were excluded, and a total of 672 patients were ultimately included (Supporting Information 1: Figure S1). With reference to the normal range established for humans (5–15 *μ*mol/L), HHcy was defined as a preoperative total homocysteine level exceeding 15 *μ* mol/L [11, 12], and patients were accordingly stratified into HHcy and normal homocysteine groups. All clinical variables were collected through a structured review of electronic medical records based on clinical expert judgment and previously reported potential influencing factors. To achieve comparability, inverse probability of treatment weighting (IPTW) was employed to balance baseline characteristics. The propensity score (PS) model included clinically relevant covariates with a standardized mean difference (SMD) > 0.1.

We first evaluated mitral valve pathology in both groups using a multimodality imaging approach incorporating echocardiography and coronary CTA to explore the potential impact of HHcy on valvular lesions. Subsequently, we conducted mid‐term follow‐up with all‐cause mortality as the primary endpoint to assess its potential effect on surgical prognosis. Secondary endpoints included early mortality, acute heart failure, acute renal failure, acute respiratory failure, stroke, malignant arrhythmia, and severe infection, with all diagnoses ascertained from patient medical records. All follow‐up was conducted through clinical visits or phone calls after discharge. The early outcome follow‐up was 30 days after discharge, and the mid‐term outcome follow‐up deadline was November 3, 2023. All procedures involving human participants in this study were in accordance with the Declaration of Helsinki (Revised 2013). The Ethics Committee of Beijing Anzhen Hospital approved the study (Institutional Review Board Document KS2022078). The ethics committee waived the need for informed consent for each patient because the study was retrospective and did not involve patient‐specific personal information.

### 2.2. Imaging Evaluation Methods

In order to better describe the differences in pathological changes of the mitral valve, we obtained preoperative echocardiography and coronary CTA images for measuring mitral valve–related indicators. All patients underwent standard echocardiography measurements using the Philips EPIQ7 intelligent color Doppler ultrasound instrument and 2–4 MHz phased array cardiac probes S5‐1 and X5‐1. In addition, all patients underwent coronary CTA examination before surgery to evaluate coronary artery disease. We analyzed the pathological changes of the mitral valve in patients using these CTA images. The Agatston scoring method on semiautomatic software (VScore, Vital Images, United States) was used to quantitatively evaluate rheumatic mitral valve calcification [13], and a threshold of 130 Hounsfield units with ≥ 3 connected pixels was used to identify calcification. Representative echocardiographic, coronary CTA images, and corresponding intraoperative findings from one patient are presented in Supporting Information 2: Figure S2.

### 2.3. Surgical Techniques

All patients underwent mitral valve repair surgery first using the standard SCORE procedure, which we have reported in detail in previous articles [14, 15]. For patients who could not undergo repair surgery after checking, we adopt mitral valve replacement surgery with preservation of the posterior leaflet and subvalvular structure.

### 2.4. Statistical Analysis

Continuous variables with normal distribution were expressed as mean ± standard deviation and compared using the *t*‐test. Continuous variables with nonnormal distribution were expressed as median (interquartile spacing) and compared using the Mann–Whitney test. The categorical variable was expressed as a number (percentage) and was compared using the chi‐square test or Fisher′s exact test as appropriate. To adjust for baseline differences between the two groups, we applied stable IPTW based on PSs. The PSs were calculated using multivariable logistic regression, with HHcy as the dependent variable and the following covariates: female, age, history of hypertension, atrial fibrillation, history of cardiovascular disease, history of cardiac surgery, NYHA functional class, and EuroSCORE II. Weights for the HHcy group were calculated using the formula PT/PS, and weights for the normal total homocysteine group were calculated using the formula [1 − PT]/[1 − PS] (P*T* is the number in the HHcy group/total number). We used the Kaplan–Meier method and log‐rank test to compare patient survival rates and plotted survival curves. We used univariable and multivariable Cox proportional hazards regression models to evaluate independent risk factors affecting patient mortality. We used a restricted cubic spline to fit the relationship between preoperative total homocysteine levels and the degree of mitral valve calcification in patients. Additionally, a Pearson correlation analysis was performed to assess the linear association between these variables. A *p* value of < 0.05 is considered statistically significant (two‐sided). All the analyses were performed with the statistical software package R (http://www.R-project.org, The R Foundation).

## 3. Results

### 3.1. Baseline Characteristics

This study included 672 patients with severe rheumatic mitral valve stenosis, and 208 (31.0%) patients had concomitant HHcy. The baseline characteristics of the two groups are shown in Table 1. After IPTW, all baseline variables were well balanced with no significant differences between the groups, ensuring good comparability (Supporting Information 3: Figure S3).

**Table 1:** Clinical characteristics.

**Variables**	**Before IPTW**			**After IPTW**		
	**Normal (** **n** = 464**)**	**Hyperhomocysteinemia (** **n** = 208**)**	**p** **value**	**Normal (** **n** = 464.48**)**	**Hyperhomocysteinemia (** **n** = 206.44**)**	**p** **value**
Age (y) (mean [SD])	58.06 (7.41)	60.06 (8.01)	0.002	58.67 (7.46)	58.27 (9.05)	0.677
Female, *n* (%)	344 (74.1)	116 (55.8)	< 0.001	317.4 (68.4)	140.0 (67.8)	0.876
Body mass index (kg/m^2^) (median [IQR])	23.53 [21.74, 25.77]	23.72 [21.54, 25.92]	0.716	23.59 [21.78, 25.81]	23.65 [21.56, 25.74]	0.982
Diabetes mellitus, *n* (%)	58 (12.5)	31 (14.9)	0.467	60.8 (13.1)	26.3 (12.7)	0.898
History of cardiovascular disease, *n* (%)	42 (9.1)	28 (13.5)	0.111	46.9 (10.1)	20.2 (9.8)	0.896
History of stroke, *n* (%)	43 (9.3)	20 (9.6)	1	41.5 (8.9)	16.8 (8.1)	0.734
History of hypertension, *n* (%)	100 (21.6)	55 (26.4)	0.196	108.9 (23.5)	51.2 (24.8)	0.731
History of chronic obstructive pulmonary disease, *n* (%)	36 (7.8)	16 (7.7)	1	38.3 (8.2)	13.2 (6.4)	0.405
History of cardiac surgery, *n* (%)	32 (6.9)	6 (2.9)	0.057	25.6 (5.5)	7.0 (3.4)	0.287
Atrial fibrillation, *n* (%)	321 (69.2)	159 (76.4)	0.067	330.0 (71.1)	145.5 (70.4)	0.874
Infective endocarditis, *n* (%)	1 (0.2)	1 (0.5)	1	0.8 (0.2)	0.6 (0.3)	0.683
NYHA function class, *n* (%)			0.336			
I	11 (2.4)	3 (1.4)		9.5 (2.1)	3.7 (1.8)	0.997
II	324 (69.8)	135 (64.9)		316.9 (68.3)	142.0 (68.7)	
III	120 (25.9)	67 (32.2)		129.4 (27.9)	57.2 (27.7)	
IV	9 (1.9)	3 (1.4)		8.4 (1.8)	3.8 (1.8)	
EuroSCORE II (median [IQR])	2.54 [1.94, 3.15]	2.58 [1.83, 3.43]	0.675	2.54 [1.91, 3.17]	2.53 [1.80, 3.41]	0.990

*Note:* Data are shown as *n* (percentage) or median (interquartile range).

### 3.2. Imaging Assessment Results

**Figure 1:**
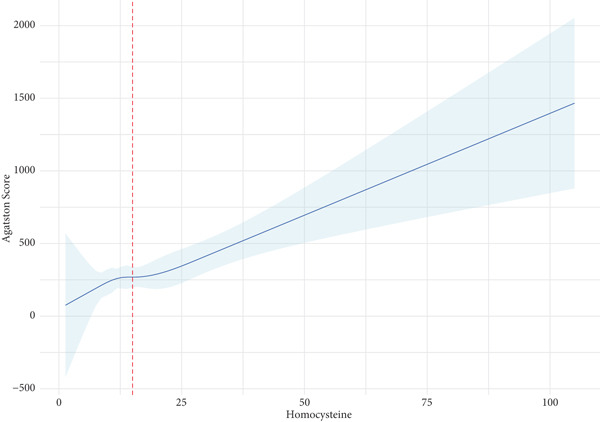
Restrictive cubic spline plot of the relationship between total homocysteine levels and mitral valve calcification Agatston score.

Echocardiography and coronary CTA were used to evaluate the characteristics of mitral valve disease in patients, and Table 2 presents the comparison results of multimodality imaging data between two groups of patients. After IPTW, patients with HHcy exhibited larger left ventricular end‐diastolic diameter (46.00 vs. 46.00, *p* = 0.044), larger left ventricular end‐systolic diameter (36.00 vs. 33.00, *p* = 0.022), higher Agatston score for mitral valve calcification (37.47 vs. 17.19, *p* = 0.038), greater mitral valve calcification quality (7.14 vs. 4.70, *p* = 0.046), and a larger volume of mitral valve calcification (32.30 vs. 20.30, *p* = 0.024). The relationship between total homocysteine levels and the Agatston score for mitral valve calcification was assessed using a restricted cubic spline (association *p* = 0.0004). The analysis revealed a positive association between total homocysteine levels and the Agatston score for mitral valve calcification, as demonstrated by restricted cubic spline analysis (Figure 1) and further supported by a significant linear correlation (Pearson′s *r* = 0.171, *p* < 0.001; Supporting Information 4: Figure S4). This dose‐dependent relationship suggests that elevated homocysteine levels may contribute to the progression of mitral valve calcification.

**Table 2:** Multimodality imaging data.

**Variables**	**Before IPTW**			**After IPTW**		
	**Normal (** **n** = 464**)**	**Hyperhomocysteinemia (** **n** = 208**)**	**p** **value**	**Normal (** **n** = 464.48**)**	**Hyperhomocysteinemia (** **n** = 206.44**)**	**p** **value**
Left ventricular end‐diastolic diameter (mm) (median [IQR])	46.00 [43.00, 50.00]	46.00 [40.00, 50.00]	0.258	46.00 [43.00, 50.49]	46.00 [39.99, 49.00]	0.044
Left ventricular end‐systolic diameter (mm) (median [IQR])	33.00 [30.00, 37.00]	36.00 [30.00, 42.00]	0.006	33.00 [30.00, 37.00]	36.00 [30.00, 43.00]	0.022
Left atrium diameter (mm) (median [IQR])	50.00 [45.00, 54.00]	51.00 [47.00, 57.00]	< 0.001	50.58 [45.00, 54.00]	51.00 [46.00, 56.07]	0.052
Left ventricular ejection fraction (%) (median [IQR])	59.00 [54.00, 64.00]	58.00 [52.00, 63.00]	0.040	59.00 [54.00, 64.00]	58.00 [52.00, 64.00]	0.104
Mean pulmonary artery pressure (mmHg) (median [IQR])	40.00 [31.00, 47.00]	42.00 [32.75, 49.00]	0.144	40.00 [30.41, 47.00]	42.00 [33.00, 49.00]	0.051
Degree of mitral stenosis, *n* (%)			0.194			0.127
None	25 (5.4)	12 (5.8)		25.6 (5.5)	10.9 (5.3)	
Mild	57 (12.3)	31 (14.9)		58.8 (12.7)	30.7 (14.9)	
Moderate	122 (26.3)	39 (18.8)		121.3 (26.1)	36.3 (17.6)	
Severe	260 (56.0)	126 (60.6)		258.5 (55.7)	128.7 (62.3)	
Degree of mitral regurgitation, *n* (%)			0.901			0.579
None	44 (9.5)	23 (11.1)		42.2 (9.1)	22.7 (11.0)	
Mild	156 (33.6)	72 (34.6)		157.0 (33.8)	78.4 (37.9)	
Moderate	99 (21.3)	43 (20.7)		95.8 (20.6)	38.7 (18.7)	
Severe	165 (35.6)	70 (33.7)		169.2 (36.4)	66.8 (32.3)	
Mitral valve calcification factors measured by cardiac CT						
Agatston score (median [IQR])	14.00 [0.00, 198.25]	44.00 [0.00, 249.50]	0.025	17.19 [0.00, 203.69]	37.47 [0.00, 210.00]	0.038
Calcification volume (median [IQR])	16.55 [0.00, 155.15]	39.30 [0.00, 235.48]	0.017	20.30 [0.00, 159.48]	32.30 [0.00, 182.77]	0.024
Calcification quality (median [IQR])	3.30 [0.00, 37.50]	9.95 [0.00, 66.65]	0.019	4.70 [0.00, 41.19]	7.14 [0.00, 43.17]	0.046
Calcification of zona pellucida, *n* (%)	58 (12.5)	28 (13.5)	0.826	58.0 (12.5)	27.2 (13.2)	0.816
Calcification penetrates, *n* (%)	59 (12.7)	35 (16.8)	0.194	62.4 (13.5)	29.5 (14.3)	0.780
Noncalcification factors measured by cardiac CT						
Length of anterior leaflets (mm) (median [IQR])	33.54 [30.89, 36.60]	33.87 [31.21, 37.03]	0.429	33.61 [31.05, 36.92]	33.10 [30.83, 36.59]	0.409
Length of posterior leaflets (mm) (median [IQR])	19.40 [17.37, 21.78]	19.12 [17.01, 21.73]	0.256	19.55 [17.39, 21.84]	19.02 [16.98, 21.40]	0.104
The thinnest part of zona pellucida (mm) (median [IQR])	0.83 [0.72, 0.96]	0.84 [0.73, 0.95]	0.781	0.83 [0.72, 0.96]	0.84 [0.73, 0.94]	0.831
The thickest part of zona pellucida (mm) (median [IQR])	1.63 [1.42, 1.90]	1.69 [1.52, 1.98]	0.008	1.64 [1.43, 1.91]	1.68 [1.50, 1.97]	0.100
Papillary muscle or chordae fusion, *n* (%)	96 (20.7)	49 (23.6)	0.463	98.4 (21.2)	48.4 (23.4)	0.538

*Note:* Data are shown as *n* (percentage) or median (interquartile range).

### 3.3. Short‐Term Outcome

After IPTW, patients with HHcy showed no significant differences in early outcomes compared to the normal group, except for a lower rate of mitral valve repair (58.7% vs. 69.8%, *p* = 0.009), as shown in Table 3.

**Table 3:** Early outcomes of patients.

**Variables**	**Before IPTW**			**After IPTW**		
	**Normal (** **n** = 464**)**	**Hyperhomocysteinemia (** **n** = 208**)**	**p** **value**	**Normal (** **n** = 464.48**)**	**Hyperhomocysteinemia (** **n** = 206.44**)**	**p** **value**
Mitral valve repair, *n* (%)	327 (70.5)	120 (57.7)	0.002	323.9 (69.8)	121.4 (58.7)	0.009
Early death, *n* (%)	3 (0.6)	4 (1.9)	0.273	2.9 (0.6)	3.4 (1.6)	0.200
Acute heart failure, *n* (%)	5 (1.1)	6 (2.9)	0.168	5.7 (1.2)	4.0 (1.9)	0.475
Acute renal failure, *n* (%)	7 (1.5)	5 (2.4)	0.621	7.0 (1.5)	3.5 (1.7)	0.830
Acute respiratory failure, *n* (%)	6 (1.3)	5 (2.4)	0.471	5.9 (1.3)	3.7 (1.8)	0.567
Stroke, *n* (%)	10 (2.2)	6 (2.9)	0.764	10.5 (2.3)	5.3 (2.6)	0.819
Malignant arrhythmia, *n* (%)	3 (0.6)	1 (0.5)	1.000	3.1 (0.7)	0.5 (0.3)	0.393
Severe infection, *n* (%)	5 (1.1)	7 (3.4)	0.079	5.0 (1.1)	5.2 (2.5)	0.141
Mechanical ventilation time (hour) (median [IQR])	19.50 [16.00, 22.00]	20.00 [16.00, 24.00]	0.050	19.50 [16.00, 22.00]	19.50 [16.00, 23.00]	0.438
ICU time (hour) (median [IQR])	21.00 [17.00, 24.00]	21.00 [18.00, 29.25]	0.043	13.00 [11.00, 16.00]	13.00 [11.00, 16.00]	0.498
Hospital stay time (day) (median [IQR])	13.00 [11.00, 16.00]	14.00 [12.00, 17.00]	0.053	21.00 [17.00, 24.00]	21.00 [17.00, 25.33]	0.390

*Note:* Data are shown as *n* (percentage) or median (interquartile range).

### 3.4. Mid‐Term Outcome

We conducted mid‐term follow‐up on the patients, with a median follow‐up time of 40.3 months. No significant difference in follow‐up time was observed between the HHcy group and the normal group (41.2 vs. 39.9 months, *p* = 0.124). During the mid‐term follow‐up, a total of 25 deaths were recorded. Of these, 14 occurred in the HHcy group and 11 in the normal group. Patients with HHcy had a significantly higher mortality rate (6.7% vs. 2.4%, *p* = 0.011). Kaplan–Meier survival analysis showed that patients with HHcy had significantly lower mid‐term survival rates (log‐rank *p* = 0.004). This finding remained consistent after IPTW (log‐rank *p* = 0.004), as shown in Figure 2. We included all preoperative factors that may affect patients′ mid‐term survival in the univariate Cox regression, and after incorporating factors with *p* < 0.05 into the multivariate regression, we found that HHcy was an independent risk factor for mid‐term survival after surgery (HR, 2.257; *p* = 0.048) (Table 4).

Figure 2Kaplan–Meier curve (a) without IPTW and (b) with IPTW. X line: follow‐up time since surgery (months). Y line: rate of survival probability.

**Figure:**
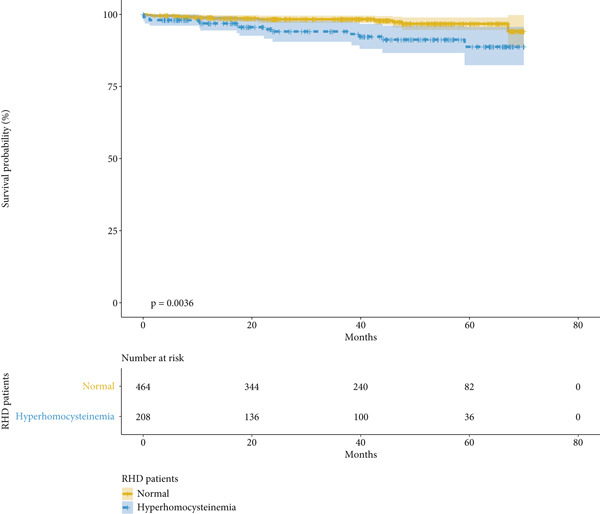
(a)

**Figure:**
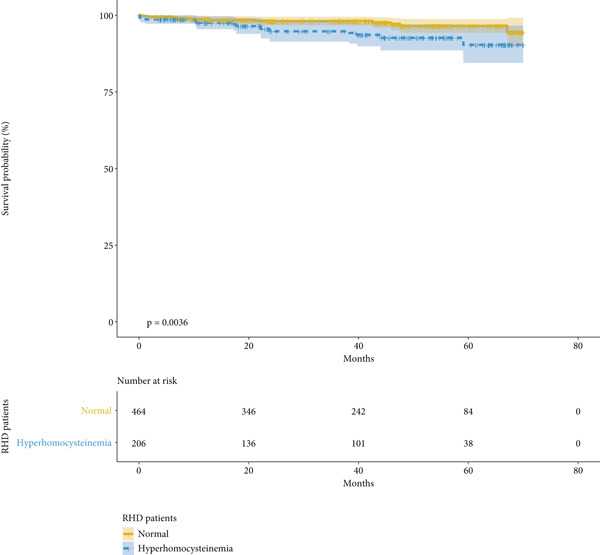
(b)

**Table 4:** Cox regression analysis of factors influencing mid‐term survival of patients.

**Variables**	**Univariate**		**Multivariate**	
	**OR (95% CI)**	**p** **value**	**OR (95% CI)**	**p** **value**
Age	1.102 (1.042–1.165)	0.001	1.097 (1.036–1.161)	0.001
Female	0.728 (0.327–1.621)	0.437		
Body mass index	0.975 (0.861–1.103)	0.684		
Diabetes mellitus	1.323 (0.494–3.545)	0.578		
History of cardiovascular disease	1.969 (0.674–5.756)	0.216		
History of stroke	2.038 (0.699–5.946)	0.192		
History of hypertension	1.016 (0.405–2.547)	0.973		
History of chronic obstructive pulmonary disease	1.042 (0.245–4.444)	0.955		
History of cardiac surgery	0.763 (0.103–5.654)	0.792		
Atrial fibrillation	3.344 (0.999–11.189)	0.051		
NYHA function class	1.458 (0.721–2.946)	0.294		
EuroSCORE II	1.111 (0.952–1.297)	0.180		
Left ventricular end‐diastolic diameter	0.974 (0.918–1.033)	0.373		
Left ventricular end‐systolic diameter	1.038 (0.997–1.081)	0.069		
Left atrium diameter	1.049 (1.011–1.089)	0.012	1.046 (1.002–1.093)	0.042
Left ventricular ejection fraction	0.993 (0.940–1.049)	0.811		
Mean pulmonary artery pressure	1.021 (1.000–1.043)	0.047	1.013 (0.991–1.036)	0.245
Degree of mitral stenosis	0.997 (0.647–1.536)	0.990		
Degree of mitral regurgitation	0.978 (0.669–1.432)	0.910		
Agatston score	1.000 (1.000–1.001)	0.436		
Calcification volume	1.000 (1.000–1.001)	0.421		
Calcification quality	1.000 (0.999–1.002)	0.642		
Calcification of zona pellucida	1.258 (0.296–5.337)	0.756		
Calcification penetrates	1.202 (0.412–3.509)	0.736		
Length of anterior leaflets	0.949 (0.869–1.037)	0.248		
Length of posterior leaflets	0.993 (0.883–1.117)	0.905		
The thinnest part of zona pellucida	0.672 (0.080–5.643)	0.714		
The thickest part of zona pellucida	1.422 (0.643–3.146)	0.384		
Papillary muscle or chordae fusion	0.651 (0.244–1.742)	0.393		
Hyperhomocysteinemia	3.051 (1.385–6.724)	0.006	2.257 (1.008–5.054)	0.048

Abbreviations: CI, confidence interval; OR, odds ratio.

## 4. Discussion

The main findings of this study in surgical patients with severe rheumatic mitral stenosis are as follows: (1) Through multimodality imaging methods to quantitatively measure mitral valve calcification in patients with rheumatic mitral valve disease, it was found that HHcy is associated with the formation of rheumatic mitral valve calcification in this surgical cohort. (2) Through univariate and multivariate Cox regression analyses, it was found that HHcy is an independent risk factor affecting the mid‐term survival of patients after surgery.

RHD remains the main cause of cardiovascular disease in young adults and the leading cause of valve disease [1, 2]. Mitral valve surgery is the most commonly performed type of surgery for patients with rheumatic valve disease [16–19]. Severe mitral valve calcification contraindicates percutaneous mitral commissurotomy, leaving surgical intervention as the only option. Moreover, in surgical patients, our previous research showed that such calcification is highly common (59% prevalence) and significantly increases the technical difficulty of mitral repair [4]. However, there is currently a lack of effective drug treatment to prevent the progression of mitral valve calcification in patients [20]. Therefore, exploring the risk factors that affect the formation of mitral valve calcification, especially those that can be intervened in advance, is of great significance.

HHcy is a common cardiovascular risk factor [7, 8]. Karger et al. first reported that HHcy may be a risk factor for the progression of severe vascular calcification [9], but so far, there has been no research reporting a correlation between homocysteine and valve calcification. In our cohort of patients undergoing rheumatic mitral valve surgery, 31% of patients were diagnosed with HHcy. However, due to a lack of research evidence, the guidelines did not mention whether these patients needed medication to reduce their total homocysteine levels.

We used a multimodality imaging method combining preoperative echocardiography and coronary CTA imaging to evaluate the correlation between total homocysteine levels and pathological changes in the mitral valve. In terms of echocardiography, we found no significant difference in the degree of mitral stenosis or regurgitation among patients, but patients with HHcy have significantly more severe left ventricular pathological remodeling. Their left ventricular end‐systolic diameter and left ventricular end‐systolic diameter were larger, which is consistent with the Framingham Heart Study [21]. Due to the limitations of echocardiography in evaluating valve calcification, we referred to the method of assessing aortic valve calcification [22, 23] and innovatively applied coronary CTA imaging of patients to evaluate mitral valve calcification, which enabled us to discover the quantitative relationship between total homocysteine levels and the degree of mitral valve calcification in patients. Our research found that patients with HHcy had significantly more severe mitral valve calcification. With a median follow‐up of 40.3 months, the Kaplan–Meier survival analysis showed that patients with HHcy had significantly lower mid‐term survival rates. Through Cox regression analysis, it was found that HHcy is an independent risk factor affecting the mid‐term survival of patients after surgery. Of note, our study found that HHcy was independently associated with both increased mitral valve calcification and postoperative mortality, as the degree of calcification itself showed no direct correlation with survival. This suggests that the adverse prognostic impact of HHcy may not be primarily mediated through its promotion of valvular calcification. Given that the surgical procedure directly removes or replaces the calcified valve, the long‐term risk is more likely attributable to the pathological states concomitant with HHcy as a systemic metabolic disorder—such as vascular endothelial dysfunction, accelerated atherosclerosis, and a prothrombotic state—all of which can contribute to long‐term cardiovascular events. Therefore, HHcy should be regarded as a comprehensive risk marker reflecting poorer overall patient health status, rather than solely a driver of local valvular calcification.

HHcy may be caused by folate or vitamin B12 deficiency [24]. Folic acid is widely used to treat HHcy, which can promote vascular health by reducing vascular oxidative stress and regulating endothelial nitric oxide synthase [25]. Liu et al. and Song et al. both found that supplementing folic acid can inhibit valve calcification. Currently, in the field of valve disease, HHcy has not received sufficient attention. This study, for the first time, found a significant correlation between HHcy and the degree of rheumatic mitral valve calcification in patients. Whether such patients have folate deficiency and whether early oral supplementation of folate or a combination therapy of folate and B vitamins can inhibit the progression of mitral valve calcification in such patients still needs further research [26]. This study is the first to demonstrate an association between HHcy and both valvular calcification and postoperative mortality in patients with rheumatic mitral stenosis. This suggests that preoperative homocysteine testing could aid in risk stratification by identifying individuals at higher risk for accelerated calcification and poorer prognosis. In patients with rheumatic mitral valve disease, such testing might facilitate closer imaging surveillance and inform earlier discussions regarding surgical timing. A critical unanswered question in therapeutic intervention is whether lowering homocysteine levels through folate or B‐vitamin supplementation in patients with established HHcy can retard the progression of mitral valve calcification or improve surgical outcomes. Although our observational design cannot directly validate the efficacy of this intervention, our findings provide a robust rationale for prospective randomized controlled trials to address this question. Furthermore, the identified link between HHcy and calcification unveils a novel avenue for basic research. Investigating whether HHcy drives calcification through mechanisms such as inducing osteogenic differentiation of valvular interstitial cells or promoting inflammation may pave the way for developing targeted therapies, offering new hope beyond surgical intervention for mitigating disease progression. In summary, our findings position homocysteine as a pivotal molecular nexus connecting clinical risk prediction, interventional target exploration, and future drug development.

This study has some limitations. First, as a single‐center retrospective study, our research cannot establish a causal relationship between HHcy and mitral valve calcification formation in patients. We cannot exclude the possibility that the progression of valvular pathology itself may lead to elevated homocysteine levels, nor can we fully rule out the potential influence of other confounding factors—such as nutritional status, medication use, or folate deficiency—on homocysteine levels. Second, while IPTW was applied to adjust for baseline differences, the potential for overfitting remains a limitation due to the sample size. Furthermore, as precise causes of death were not available, all‐cause mortality was used as the endpoint, which may obscure specific cardiovascular associations. Additionally, our findings are limited to surgical patients and may not generalize to all RHD populations due to selection bias. Finally, our study lacked mechanism exploration and could only suggest the correlation between HHcy and mitral valve calcification and poor prognosis, without providing evidence for further intervention targets. Our research is still ongoing, and in the future, we will explore whether drug treatments that reduce total homocysteine levels can benefit patients with rheumatic valve disease.

## 5. Conclusion

HHcy was associated with the formation of rheumatic mitral valve calcification and increased mid‐term postoperative mortality. Further research is needed to determine whether the treatment of reducing total homocysteine levels can benefit patients with rheumatic mitral valve disease.

**Nomenclature**
RHD: rheumatic heart disease
IPTW: inverse probability of treatment weighting
HHcy: hyperhomocysteinemia
BMI: body mass index
CTA: computed tomography angiography
MPR: multiplanar reconstruction
PBMC: percutaneous balloon mitral commissurotomy
NYHA: New York Heart Association
IQR: interquartile range
OR: odds ratio
CI: confidence interval

## Ethics Statement

All procedures involving human participants in this study were in accordance with the Declaration of Helsinki (Revised 2013). The Ethics Committee of Beijing Anzhen Hospital approved the study (Institutional Review Board Document KS2022078). The ethics committee waived the need for informed consent for each patient because the study was retrospective and did not involve patient‐specific personal information.

## Disclosure

All authors read and approved the final manuscript.

## Conflicts of Interest

The authors declare no conflicts of interest.

## Author Contributions

S.J. and P.L. contributed equally to this work. S.J., P.L., Hk.Z., T.L., X.Y., R.J., X.H., Y.H., L.X., X.M., Ma.W., X.Z., W.L., Me.W., Hj.Z., and W.J. devised the study concept and designed the study. S.J., P.L., Ma.W., X.Z., W.L., Me.W., Hongj.Z., and W.J. conducted the study. S.J., P.L., Ma.W., and W.L. conducted the data collection and analysis and drafted the manuscript.

## Funding

This study was funded by the Noncommunicable Chronic Diseases‐National Science and Technology Major Project (2023ZD0514000), the National Key Research and Development Program of China (10.13039/501100012166) (2021YFC2501104 and 2022YFE0209800), the National Natural Science Foundation of China (10.13039/501100001809) (82422007, 82241205, and 82170487), the Beijing Municipal Natural Science Foundation (10.13039/501100005089) (JQ24039 and 7244326) and the Beijing Anzhen Hospital Major Science and Technology Innovation Fund (KCZD202203 and KCQY202201).

## Supporting Information

Additional supporting information can be found online in the Supporting Information section.

## Supporting information


**Supporting Information 1** Figure S1: Study design.

**Supporting Information 2** Figure S2: Multimodality imaging evaluation and intraoperative findings in the same patient. (A) Short‐axis view on coronary computed tomography angiography (CTA). (B) Long‐axis view on coronary CTA. (C) Intraoperative view of the mitral valve. (D) Transthoracic echocardiogram.

**Supporting Information 3** Figure S3: Covariate balance.

**Supporting Information 4** Figure S4: Linear correlation between total homocysteine levels and mitral valve calcification burden. Scatter plot showing the association between preoperative homocysteine levels and Agatston scores quantified by cardiac CT. The solid red line represents the linear regression fit, with the shaded area indicating the 95% confidence interval. The Pearson correlation coefficient (*r*), *p* value, and linear regression equation are annotated on the graph.

## Data Availability

Data is available upon request from the authors.
