# Supplementary material for: Impact of Hyperhomocysteinemia on Valve Calcification and Prognosis in Rheumatic Mitral Valve Surgery
Source: Cardiovasc Ther. 2025 Dec 12;2025:5833541. doi: 10.1155/cdr/5833541 (PMC12721752; doi:10.1155/cdr/5833541)
Supplement: Supplementary file 1 — Supporting Information 1 Figure S1: Study design. [file CDR-2025-5833541-s002.pdf]

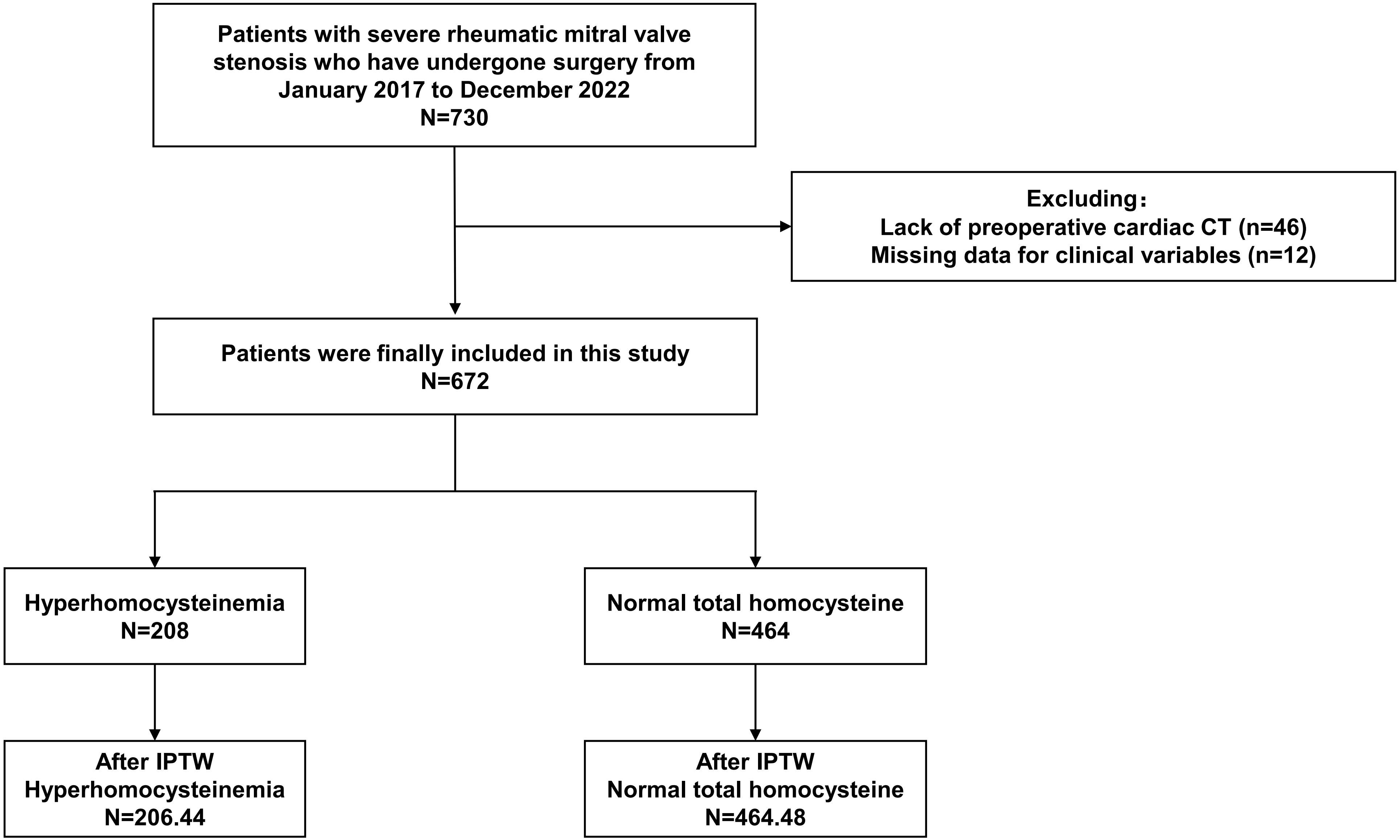
Patients with severe rheumatic mitral valve stenosis who have undergone surgery from January 2017 to December 2022
N=730
Excluding：
Lack of preoperative cardiac CT (n=46)
Missing data for clinical variables (n=12)
Patients were finally included in this study
N=672
Hyperhomocysteinemia
N=208
Normal total homocysteine
N=464
After IPTW
Hyperhomocysteinemia
N=206.44
After IPTW
Normal total homocysteine
N=464.48

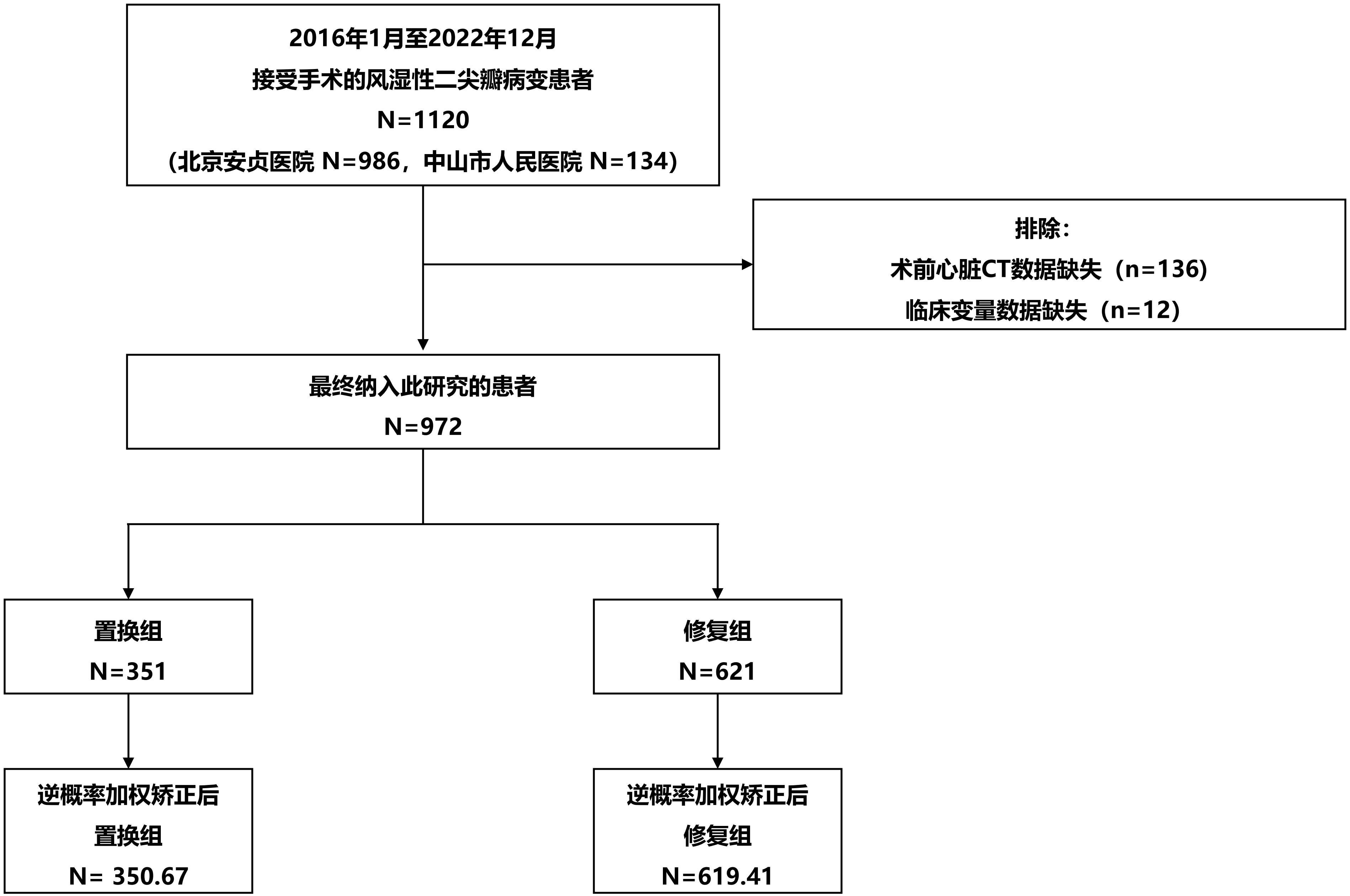
2016年1月至2022年12月
接受手术的风湿性二尖瓣病变患者
N=1120
(北京安贞医院 N=986，中山市人民医院 N=134)
排除：
术前心脏CT数据缺失（n=136)
临床变量数据缺失（n=12)
最终纳入此研究的患者
N=972
置换组
N=351
修复组
N=621
逆概率加权矫正后
置换组
N= 350.67
逆概率加权矫正后
修复组
N=619.41

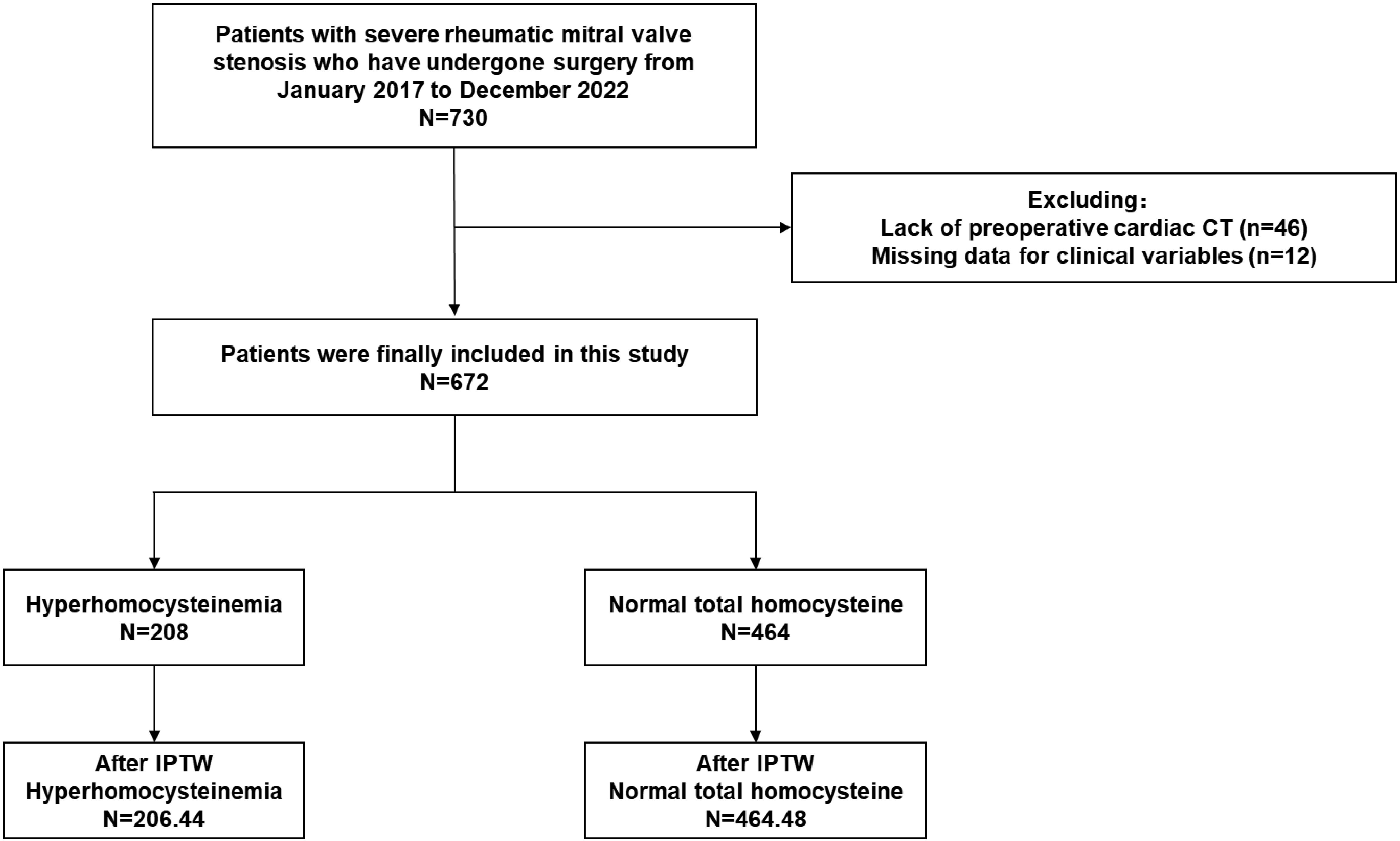
Patients with severe rheumatic mitral valve stenosis who have undergone surgery from January 2017 to December 2022
N=730
Excluding：
Lack of preoperative cardiac CT (n=46)
Missing data for clinical variables (n=12)
Patients were finally included in this study
N=672
Hyperhomocysteinemia
N=208
Normal total homocysteine
N=464
After IPTW
Hyperhomocysteinemia
N=206.44
After IPTW
Normal total homocysteine
N=464.48
