# Supplementary figures and images for: Impact of Hyperhomocysteinemia on Valve Calcification and Prognosis in Rheumatic Mitral Valve Surgery
Source: Cardiovasc Ther. 2025 Dec 12;2025:5833541. doi: 10.1155/cdr/5833541 (PMC12721752; doi:10.1155/cdr/5833541)

A

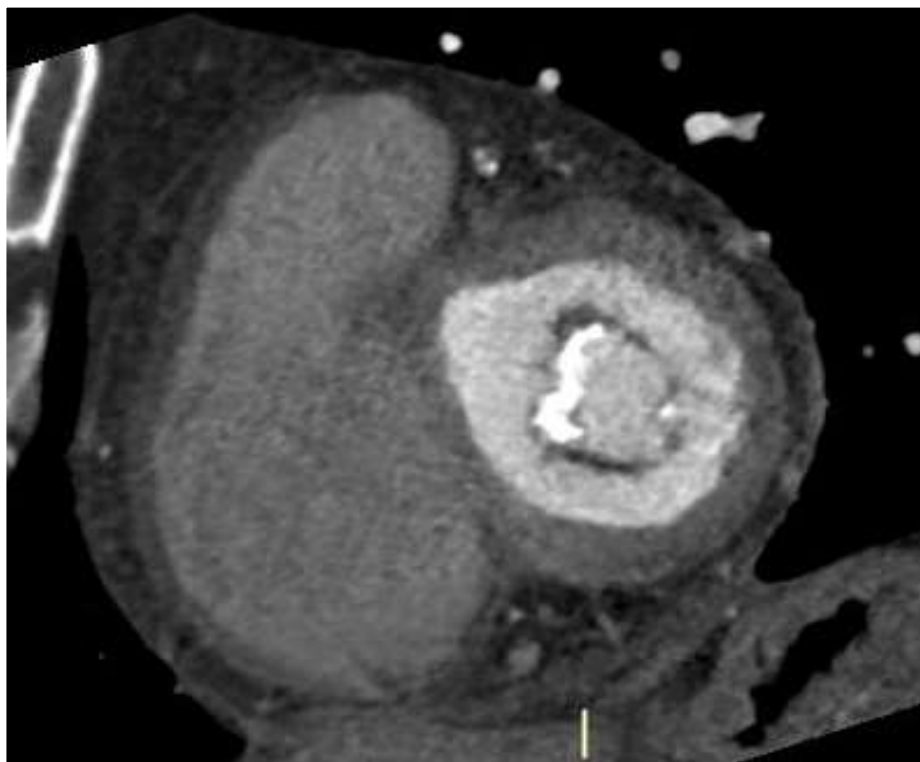

B

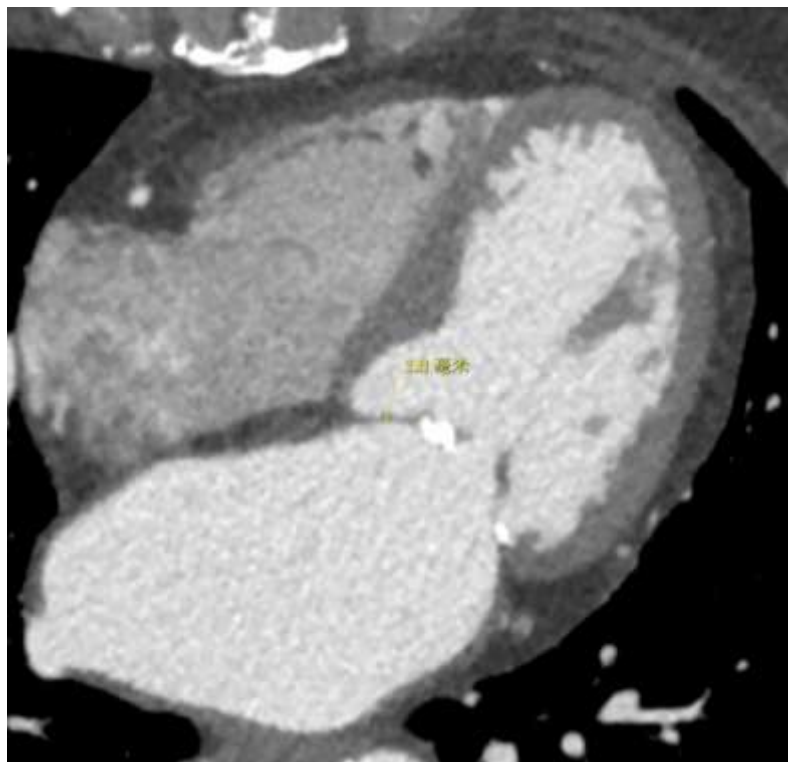

C

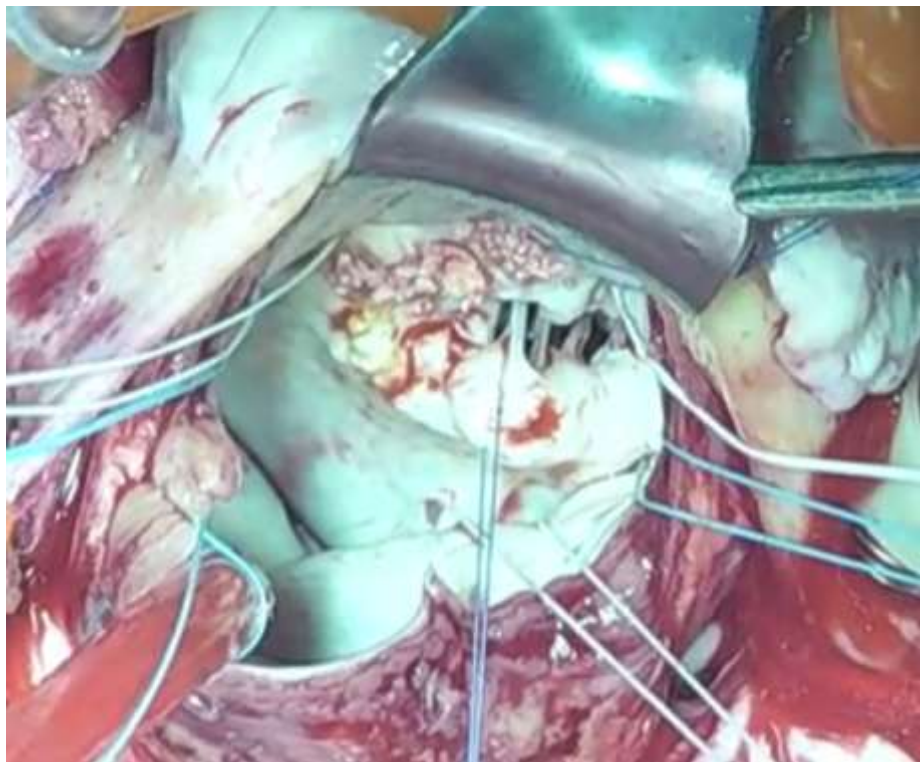

D

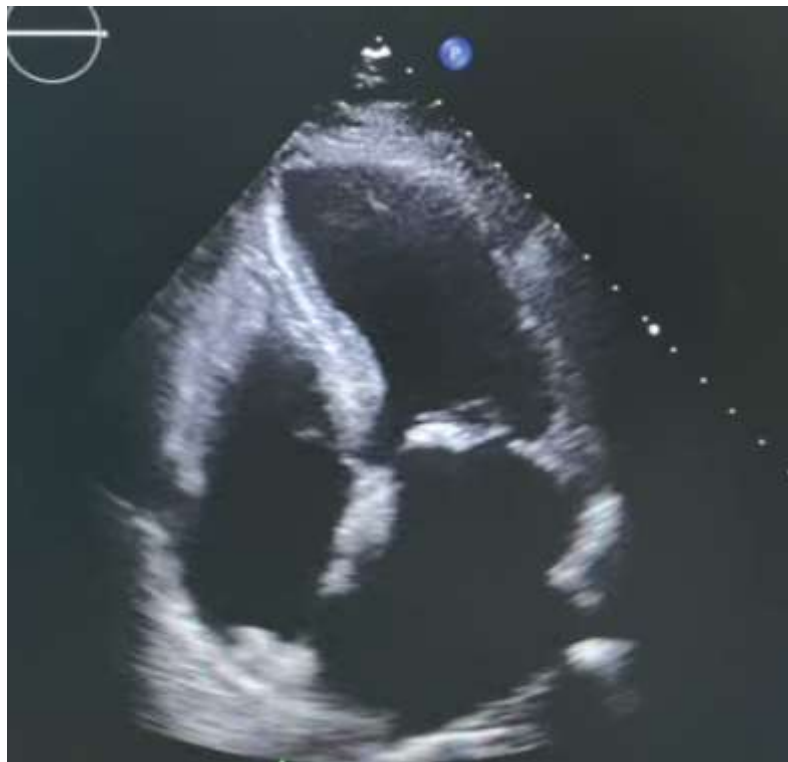

Supplement: Supplementary file 2 — Supporting Information 2 Figure S2: Multimodality imaging evaluation and intraoperative findings in the same patient. (A) Short‐axis view on coronary computed tomography angiography (CTA). (B) Long‐axis view on coronary CTA. (C) Intraoperative view of the mitral valve. (D) Transthoracic echocardiogram. [file CDR-2025-5833541-s003.pdf]

variable

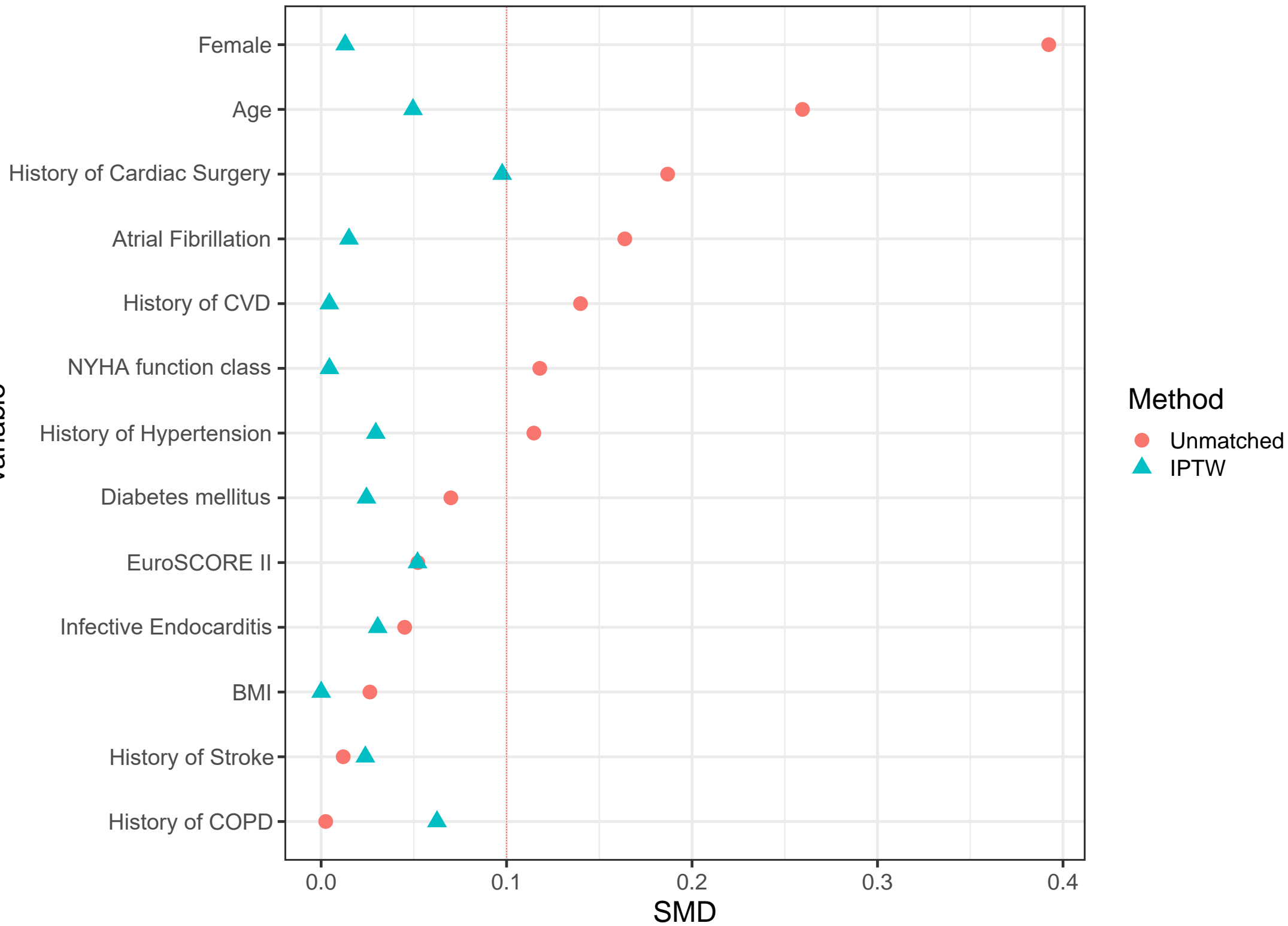

Supplement: Supplementary file 3 — Supporting Information 3 Figure S3: Covariate balance. [file CDR-2025-5833541-s001.pdf]

# Correlation between Homocysteine Levels and Mitral Valve Calcification

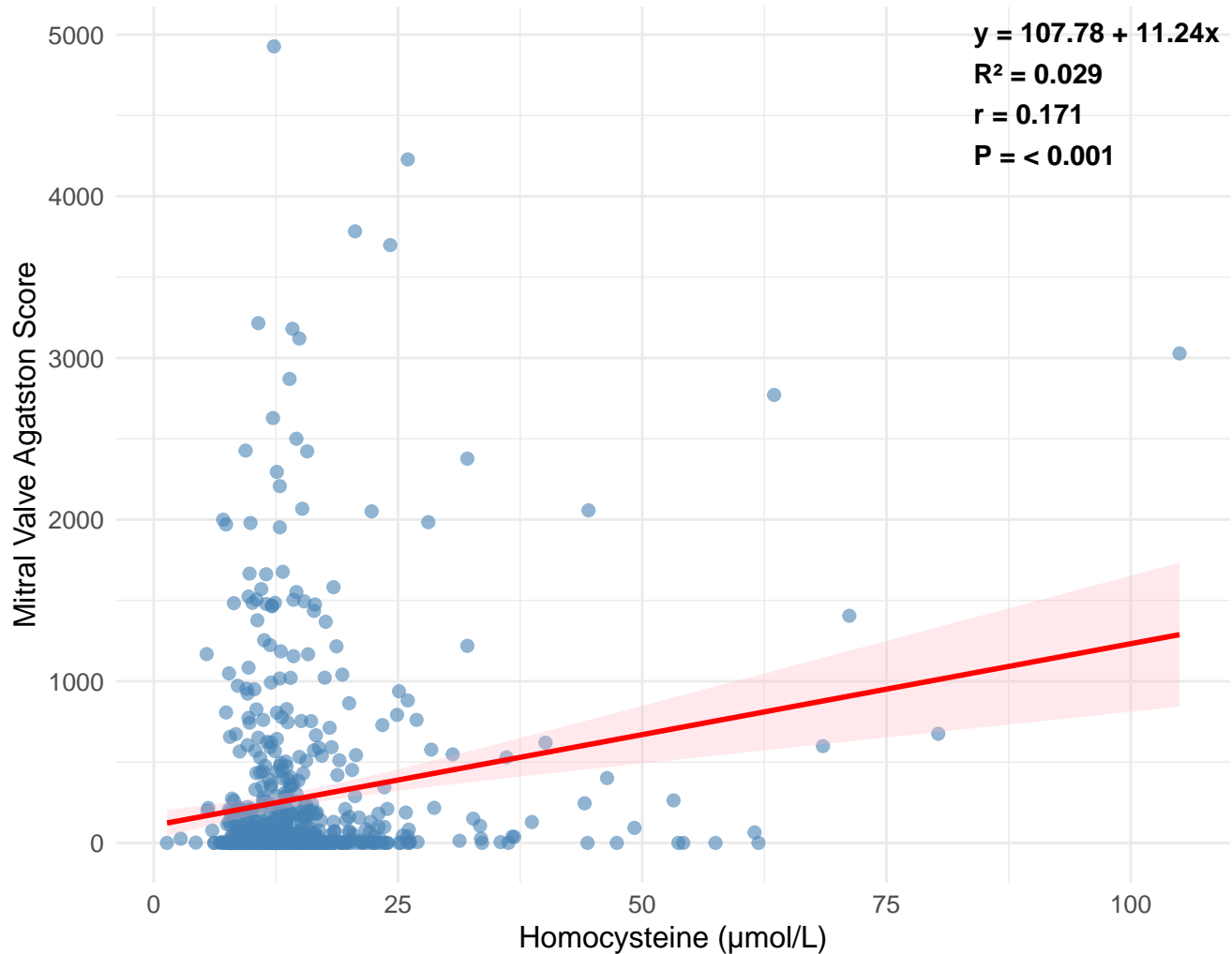

Supplement: Supplementary file 4 — Supporting Information 4 Figure S4: Linear correlation between total homocysteine levels and mitral valve calcification burden. Scatter plot showing the association between preoperative homocysteine levels and Agatston scores quantified by cardiac CT. The solid red line represents the linear regression fit, with the shaded area indicating the 95% confidence interval. The Pearson correlation coefficient (r), p value, and linear regression equation are annotated on the graph. [file CDR-2025-5833541-s004.pdf]
